# Beyond policy and planning to practice: getting sexual health on the agenda in Aboriginal communities in Western Australia

**DOI:** 10.1186/1743-8462-5-3

**Published:** 2008-05-19

**Authors:** Sandra C Thompson, Heath S Greville, Rani Param

**Affiliations:** 1 Centre for International Health, Curtin University of Technology, Perth, Western Australia, Australia; 2 Sexual Health and Blood-borne Virus Program, Department of Health, Perth, Western Australia, Australia; 3 Centre for Developmental Health, Curtin University of Technology and the Telethon Institute for Child Health Research, Perth, Western Australia, Australia

## Abstract

**Background:**

Indigenous Australians have significantly poorer status on a large range of health, educational and socioeconomic measures and successive Australian governments at state and federal level have committed to redressing these disparities. Despite this, improvements in Aboriginal health status have been modest, and Australia has much greater disparities in the health of its Indigenous people compared to countries that share a history characterised by colonisation and the dispossession of indigenous populations such as New Zealand, Canada and the United States of America. Efforts at policy and planning must ultimately be translated into practical strategies. This article outlines an approach that was effective in Western Australia in increasing the engagement and concern of Aboriginal people about high rates of sexually transmissible infections and sexual health issues. Many aspects of the approach are relevant for other health issues.

**Results:**

The complexity of Indigenous sexual health necessitates inter-agency and cross-governmental collaboration, in addition to Aboriginal leadership, accurate data, and community support. A recent approach covering all these areas is described. This has resulted in Aboriginal sexual health being more actively discussed within Aboriginal health settings than it once was and additional resources for Indigenous sexual health being available, with better communication and partnership across different health service providers and sectors. The valuable lessons in capacity building, collaboration and community engagement are readily transferable to other health issues, and may be useful for other health professionals working in the challenging area of Aboriginal health.

**Conclusion:**

Health service planners and providers grapple with achieving Aboriginal ownership and leadership regarding their particular health issue, despite sincere concern and commitment to addressing Aboriginal health issues. This highlights the need to secure genuine Aboriginal engagement. Building capacity that enables Indigenous people and communities to fulfill their own goals is a long-term strategy and requires sustained commitment, but we argue is a prerequisite for better Indigenous health outcomes.

## Introduction

Discussions of Aboriginal and Torres Strait Islander health generally acknowledge the many areas of disadvantage experienced by Australia's Indigenous people, reflected in measures of education, employment, income and housing [1]. It is also well known that poverty and life stresses interfere with the capacity of people to prioritise health above other pressing matters which have more immediate impact on survival and living. Indigenous Australians suffer disproportionate rates of sexually transmitted infections (STI) compared to the general population [2] and these high rates of bacterial infections have persisted despite being curable with short courses of antibiotics. In addition, STIs have extra importance as a surrogate marker of HIV risk [3].

We argue that, for a long time, STI control in Western Australia was one health issue (among many) that deserved to be prioritised for effective action by Aboriginal people, supported by policy and resources. By the second half of the 1990s, there was also increasing recognition that high rates of STIs have many causes and are not simply the result of promiscuous sexual behaviour. In particular, high rates reflected deficiencies in access to, and the nature of, services. This recognition signaled a new era where, in addition to efforts that had been directed at promoting safe sex [4], increasing efforts were directed at the provision of appropriate and comprehensive primary health care. Yet until recently, most efforts to address STI control were driven by non-Aboriginal people, and disease rates have remained refractory to well-intentioned interventions. Efforts, mainly by non-Aboriginal people to engage Aboriginal people in policy, planning and review of service aspects, had largely failed, so that often discussions of Aboriginal health issues were occurring without Aboriginal community members or health professionals in attendance. Appropriate engagement strategies that go beyond extending an invitation to an "identified" Aboriginal representative to attend meetings is required if genuine engagement is to occur.

While STI rates remain high in Aboriginal people in WA, those engaged in planning and service delivery for sexual health note a shift from the recent past of lamenting the lack of Aboriginal involvement in STI issues, to the present where there are many Aboriginal advocates for a comprehensive approach that includes better education and clinical services. Recent conversations with non-Aboriginal colleagues working on other health issues where Aboriginal people suffer disproportionate morbidity and mortality indicate that they are experiencing the same frustration that we experienced working in sexual health a few years ago; we struggled to find a way to improve the appropriateness of our practices and approaches in order to engage meaningfully with Aboriginal people to support communities in addressing priority health problems.

Some of the approaches and lessons we learned from our journey in building Aboriginal capacity to get sexual health issues on the agenda can be readily translated and may be useful for others working in Aboriginal health. The ongoing poor state of Aboriginal health requires that we reflect upon the issue of how to move from policy into practice. While working in program areas of State and Australian Government public service, we did not consciously follow a methodology based upon community development and capacity building. However, as the following outline reveals, our processes encompassed the five elements of capacity building that we subsequently realised that Garlick had earlier identified: knowledge building; leadership; network building; valuing community and their capacity to work together to achieve their own objectives; and supporting the capacity to collect, access and utilise quality information [5]. Our journey illustrates some practical examples of these elements.

### Leadership: prioritising Indigenous sexual health at the program level

From 1995–1997, a review of sexual health services within WA involving extensive statewide consultation with the Aboriginal community resulted in a document given the unlikely name of the Explicit Performance Standards (EPS). The EPS process was finalised in March 1997 and made key recommendations relating to workforce training, community education and quality management of STDs and HIV [6]. It identified Aboriginal sexual health as a high priority, providing a policy blueprint for actions to improve programs in delivery of sexual health services with a specific focus on Aboriginal people. The process of undertaking the review was costly both in terms of departmental resources and the non-remunerated inputs of time and energy of Aboriginal community members. Despite promises for increased resources for the 10 year strategy [7], sustained and substantial funds to action this blueprint did not eventuate, angering those who were aware of the costs of the consultation, and particularly the unfortunate consequences of broken promises. Nevertheless, given the public declaration of the policy, it provided useful leverage for action.

A key principle of public health is to reduce health inequalities. It was important to make Aboriginal STI control a core priority and a shared value in policy, purchasing, training activities and program delivery. An independent review of all the non-government organisations funded by the Sexual Health and Blood-borne Virus Program (SHBBVP) was undertaken, and the Program used that to rationalise future contracting of services. For example, as a purchaser of services, the SHBBVP required that Aboriginal sexual health and/or BBVs were specifically addressed in the business plans and contracts for services of all of the non-government organisations for which we had contracting responsibility, regardless of whether they had previously had any focus on Aboriginal sexual health. Similarly, we required outputs and reporting from public sector area health services in performance contracting and monitoring. This could be considered equating to Garlick's leadership criteria by providing an enabling environment for reorientation of services.

Staff at the SHBBVP unanimously recognised the need to make Aboriginal sexual health and blood-borne virus issues a priority. Shared values and commitment meant discussion within the team concentrated upon how, and not whether, to enhance opportunities for STI/BBV programs with Aboriginal people. Some staff had worked with, and built relationships with, Aboriginal people over a long period of time. It was accepted that improvements required long-term commitment and building capacity in the sector – both for Aboriginal and non-Aboriginal workers. The team worked with the Office for Aboriginal and Torres Strait Islander Health in the Australian Government Department of Health and Ageing to fund specific Aboriginal programs within some non-government organisations. As a result of sustained efforts, we eventually received in 2000 one-off additional funding to help support implementation of EPS, making possible the funding of innovative programs with dedicated positions to address Aboriginal sexual health.

### Promoting a comprehensive population health approach

Organisations were encouraged to adopt a comprehensive approach to STI control and the eight-way strategy originally advocated in Australia by Nganampa Health Services was promoted to those delivering sexual health services in Aboriginal communities [8]. This reminded those involved in STI control that it was not enough to focus on delivery of clinical services, or training or health promotion, or even all three. Although these three things were vital, other essential components were surveillance of disease and for risk behaviours, planning and management, evaluation and research, and ready access to the technical means to reduce transmission (for example, condoms, needles and syringes and sterile equipment for ceremonial purposes).

### Aboriginal leadership

Committees are inevitable in the administration of complex human services, but they can consume time without effectively achieving outcomes. The WA Indigenous Sexual Health Advisory Committee was initiated to provide a forum for stakeholders to meet and develop priorities. Members initially appointed to represent an organisation but who rarely attended were gradually replaced by those with a passion to make a difference in this area. Corresponding with this was an increase in attendance by Aboriginal members and greater stability and continuity of membership. This was helped by the Committee being not just a forum for discussion but also having decision-making power given its role in allocating the WA funding available under the National Indigenous Australians' Sexual Health Strategy. Advocacy and leadership were also demonstrated by another mainstream WA sexual health advisory committee, with an active chairperson willing to personally ensure that Aboriginal sexual health issues were brought to the attention of the Director General of Health.

A field placement of an Aboriginal epidemiology student within the Sexual Health and Blood-Borne Virus program enabled an Aboriginal health professional to build, over time, in-depth knowledge and interest in disease surveillance and the epidemiology of STIs/BBV. It helped create a space in which debate about approaches within Aboriginal health in WA was legitimized, and for concern to lead to action. A student was able to work with stakeholders external to the Department in a way that was not as constrained as that of employees within the public service. Moreover, the importance of an Aboriginal person being an authoritative source of information about STIs within the Indigenous community cannot be overestimated. There was now a champion raising the issue of STI/HIV with other key Aboriginal leaders and professionals. With information solidly grounded in data, an Aboriginal advocate was able to convey concern, outrage, and the need for united action to Aboriginal colleagues and key non-Aboriginal bureaucrats and politicians. Moreover, by framing the current high rates of disease within a human rights context, the individual concerned successfully exhorted other key Aboriginal leaders to talk about STIs/HIV and in turn become advocates for more effective responses. These new voices joined those of non-Aboriginal others who had been vigorously arguing for more commitment and resources from government to ensure that the EPS recommendations could be implemented. The benefits that derived from an Aboriginal person working within the sexual health program area in the Department of Health were readily apparent and the program staff learnt much from his perspective and those of other Aboriginal people enlisted by him for support. Ultimately, the momentum generated helped achieve approval of the creation of two dedicated Aboriginal sexual health positions within the SHBBVP.

### Supporting the capacity to collect, access and utilise quality information

In 1999 there was little detailed analysis of STI notification data in WA. Early in the process of raising interest, we were told by some public servants that the high rates of infection in Aboriginal communities were a matter of shame, that people did not want to know and that making available epidemiological information on infection rates by Aboriginality risked further stigmatising Aboriginal people. However, early workshops and meetings we had with Aboriginal people suggested otherwise. Unanticipated media publicity increased the level of interest. For example, a report on a meeting to plan training for health care providers in Aboriginal communities to deal with disclosures of child sexual abuse was quoted in an Australian Broadcasting Corporation Four Corners report and the report linked on their website [9,10]. Information on rates of STIs in Aboriginal people and minors was then subpoenaed for a coronial inquest into the death of a 15 year old Aboriginal girl who had made allegations of physical and sexual abuse prior to being found hanging in a disused toilet block [11,12]. The Coroner's findings were the catalyst for the Premier of Western Australia to commit the government to a three-member inquiry into child abuse and family violence in Aboriginal communities. All of this increased awareness among Aboriginal people that the Aboriginal community had much higher rates of sexually transmitted infections. As these conversations occurred, the response from Aboriginal people was typically expressed as: "Why hasn't anyone told us this before?" Encouraged by these responses, we continued to analyse the data by Aboriginal status and use it to demonstrate the substantial health inequalities [3,13]. This shifted the debate from concerns with shame – and yet another depressing Aboriginal health statistic – to indignation. Importantly, the issue had been re-framed in terms of a human rights agenda.

High rates of STIs are a consequence of many things, but the substantially higher rates in remote areas almost certainly reflected poorer access to appropriate services. Moreover, with a spike in Aboriginal HIV infections and an outbreak of syphilis in one WA health region that continued for over 12 months [14], there was good reason for concern to mount. Information initially developed for PowerPoint presentations was printed in colour on A4 sheets and laminated. These 'placemats' included both graphs showing rates and relative rates of disease in Aboriginal and non-Aboriginal people and key messages. They provided a format that was easy to read, readily transportable, easily usable and less readily disposed of. This was a particularly successful method of engaging with senior bureaucrats, health professionals and Aboriginal leaders and communities.

### Knowledge building

In addition to disseminating information from existing data collections, where there were gaps in our understanding, we sought to ensure that additional knowledge was created and made available. In addition to the needs assessments previously mentioned, we recognised limitations in our knowledge around injecting drug use in Aboriginal people in Western Australia and the experiences and needs of Aboriginal people with HIV. A research project to examine the harm reduction needs of Aboriginal people who inject drugs was tendered out and a detailed examination of the issue provided data and recommendations to support further efforts at harm reduction to government and Aboriginal organisations and leaders [15]. We advocated for and were involved in research to learn more about Aboriginal people living with HIV in Western Australia. To ensure proper processes of engagement, and given the sensitivity of HIV within the Aboriginal community, the research was supported by a Project Reference Group and a Steering Committee. Through the involvement of five Aboriginal members drawn from health and research backgrounds and representatives from the HIV positive Aboriginal community, the research brought many stakeholders together to exchange views and enabled the voices of Aboriginal people with HIV to inform policy and services [16]. The report highlighted the importance of dealing with substance use, housing, and transport so that Aboriginal people could engage with their health issues. Systematically collected information added weight to existing concerns about the need for more responsive services and led to changes in the model for service delivery in Perth.

The EPS review had identified the need for workforce development, especially targeting undergraduate medical students, remote area nurses and Aboriginal Health Workers. Training needs analyses were undertaken to identify specific areas for professional development and in response there were successful efforts to increase the emphasis on sexual health and on Aboriginal health in the curriculum of relevant training programs.

In an effort to develop special expertise in the Aboriginal workforce, emphasis was put on building knowledge and capacity, and the approach adopted was to have Aboriginal staff working side-by-side with experienced sexual health practitioners. This was most readily achieved within well-managed organisations committed to supporting individuals and to improving Aboriginal health. It enabled mutual sharing of knowledge and fostered the development of understanding and respect. An example of this was the development of a very successful training program developed in FPWA (formerly Family Planning WA), that provided Indigenous Trainees one or two years of apprenticeship style training. Establishment of Aboriginal positions in other organisations such the WA AIDS Council, the Hepatitis Council and Aboriginal trainee positions in the drug and alcohol sector assisted building a critical mass of Aboriginal workers networking in sexual health. Building working partnerships between Aboriginal and non-Aboriginal professionals provided mutual benefits and built better cross-cultural understanding.

Provided with encouragement, organisations developed creative ideas into novel programs and sought additional funding through competitive processes. For example, FPWA developed an innovative education program which enabled sexual health education to be delivered in remote communities by peer educators. Using a train-the-trainer approach, the program targeted community workers in sectors such as justice, education, youth and community development, in addition to health. Moving outside the health sector underscored the contribution that multiple sectors with an interest in Aboriginal issues could have in addressing Aboriginal sexual health, improved partnership between sectors, enhanced skills in the Indigenous community workforce, and increased the number of Aboriginal people advocating for improvements to Indigenous sexual health.

### Be opportunistic as well as strategic

Health operates in a political landscape and while strategic approaches are essential, it is important to make the most of opportunities that may not have been anticipated. A new Australian government initiative, the Donovanosis Eradication Strategy [17], led to the appointment of a Donovanosis Project Officer who maintained interest in clinical services and health promotion in Aboriginal sexual health. She supported and up-skilled remote practitioners, traveling extensively to provide on-site training and practical support for system improvements. Close contact with practitioners enabled her to gather intelligence about common issues and local problems that could be fed back into planning, funding and support. A refractory outbreak of syphilis in a regional area increased the number of people actively lobbying senior bureaucrats in the WA Department of Health and the WA Minister of Health for the additional resources needed for more effective action. As discussed earlier circumstances which led to a government inquiry into the response of government agencies to sexual abuse and family violence in Aboriginal communities [18] received considerable media attention, providing another opportunity to highlight the disproportionate rates of STIs in Aboriginal people, particularly in young adolescents.

### Developing appropriate resources

For many years, in the course of their professional work the authors had often encountered comments about the dearth of appropriate resources for promoting sexual health in Aboriginal communities in WA. The absence of appropriate materials appeared to underpin a reticence to engage in sexual health education and promotion of sexual health messages in the community. "Mission Possible", an offshoot of the Donovanosis Elimination Project, brought together a small group of Aboriginal and non-Aboriginal health promotion practitioners with a commitment to improvements in sexual health. The group collectively set goals for resource production and maintained momentum and enthusiasm. The process of meeting regularly with a common purpose to develop the resources (brochures and flip charts for use with males and with females) was important. A range of new resources was ultimately printed and widely distributed by the WA Department of Health, further helping health workers to engage with and undertake education within Aboriginal communities.

### Network building

Health professionals working in remote and rural areas often feel a sense of isolation exacerbated by the need for strict medical confidentiality in small country towns. In addition to sharing ideas and building skills within organisations, it was clear that we also needed accelerate knowledge building and networking between organisations [5]. Following a tender process, FPWA was funded to support the development of a statewide sexual health network maintained through regular e-mail and teleconferences and an annual face-to-face Forum. This provided professional development opportunities and enhanced learning through sharing problems and stories of success. For many Aboriginal health workers and rural and remote non-Indigenous workers, opportunities to talk about their work in sexual health had been limited. Each annual Forum was attended by health professionals within the many disciplines that contribute to public health, and provided opportunities for professional development, validation of the importance of their role, peer-support, and the chance to share their ideas and learn about initiatives elsewhere. As the momentum increased, Aboriginal people assumed greater responsibility for planning and delivering the Forum program. In addition to the Forum and network, special training workshops directed at providers delivering services in Aboriginal communities were held on specific issues such as managing HIV, hepatitis C, and dealing with child sexual abuse. The opportunity for various workers and agency representatives to meet regularly both face-to-face and by teleconference helped build relationships across all areas of the sector.

Promoting Indigenous sexual health in State and National research projects provided further data and information, and generated more interest and momentum beyond the sexual health sector. There were now a number of organisations and individuals who were actively taking opportunities in their professional and personal lives to advocate and lobby for more resources and effort to address the longstanding high rates of STIs in Aboriginal people in WA through better education and services. Instead of limiting our efforts to assorted specialties within Health, areas of common concern were highlighted outside of the health arena, including with Indigenous Affairs, and the youth and welfare sectors. This cross-governmental, inter-agency collaboration contributed substantially to a more focused and strategic effort. An ad hoc multisectoral, non-departmental working group of interested Aboriginal and non-Aboriginal public servants, academics and service providers began a concerted push for additional resources and commitment. The group called on the Minister for Health to attend a Summit on Aboriginal Sexual Health and to respond to resolutions from the event.

### Valuing Aboriginal community and their capacity to work together to achieve their own objectives

Efforts over four years to gain political attention and build capacity which incorporated all the elements of Garlick's framework [5] culminated in a Summit entitled *Moving Beyond Shame: Information, Partnership and Action on STIs*. The Summit brought together Aboriginal people from across the state to highlight concerns and create an agenda for action. The Summit successfully gained political attention and the commitment of additional resources to address sexual health in rural and remote areas. The placement in three remote regions of teams of three working in both public sector community health services and Aboriginal community-controlled health services has continued to build capacity, communication and partnerships within and across regions with high rates of Aboriginal STIs.

The first Western Australian Aboriginal Sexual Health Strategy was developed in 2005, outlining a comprehensive approach to primary health care and population health to improve sexual health indicators for Aboriginal people living in WA [19]. The Strategy followed broad consultation with stakeholders throughout Western Australia, led by an Aboriginal man, and is based upon three principles: partnerships and comprehensive programs, proactive community responses, and primary health care approaches. As disease control is the ultimate aim and because the systems exist to collect and analyse STI data, the success or failure of the strategy will inevitably be judged by changes in the burden of STIs in the Aboriginal population. However, in a liberal democracy such as Australia, we are convinced that success is more likely with the support of a broad range of Aboriginal people and communities who have the knowledge, interest and standing in their communities to tackle issues which only a few years ago were unknown or simply low priority, although this is something less readily measured.

## Conclusion

We acknowledge that this is an abbreviated and partisan description of a process that has begun but is by no means completed, and that an objective measure of success, reduced disparity in STI rates, is still to be achieved. However, we believe that there are many lessons that we have learned around engaging with Aboriginal communities that could be useful for those involved in health policy and planning with marginalised groups. These key lessons learned (see Box) might be helpful for others struggling to build capacity, achieve engagement with vulnerable groups and advocate for greater attention to their health issues. On reflection, there is an inevitable challenge between the holistic approach that Indigenous Australians want in relation to health, and the "body part" or systems approach that is often the way in which policy and program funding operates.

The approach described operated statewide but incorporates important elements of community development approaches that have been described for projects at the local level [20-22]. These include building trust, education and training to develop awareness, skills and leadership. Particularly important is the employment of Aboriginal staff with provision for peer and management support for Aboriginal staff and modeling good work practices by locating Aboriginal positions within effective and well managed organisations which enables two way learning. Other aspects were networking to support isolated practitioners; creating fora to provide a safe and enjoyable space for vigorous discussion and exchange of views; leadership from Aboriginal people and extracting public commitments to partnership and action. Aboriginal people need to lead their own development in terms of sexual health but this requires support from all health professionals. Enabling this to occur has implications for policy and planning. Given the need for reporting and measurement of outcomes, a challenge for public health practitioners, program managers and policy makers in an era where "capacity building" has become common parlance is to develop tools to measure the increased capacity, and to demonstrate that building capacity does translate into, and is a prerequisite for, better health outcomes. "Capacity" is not a commodity to be purchased but results from sustained efforts at development and engagement over time. Measures of capacity might be around that Aboriginal engagement: for example, Aboriginal knowledge and education about a health issue; improved representation of Aboriginal people on committees with power to influence planning and resourcing; or increased roles for Aboriginal people in setting the agenda in relevant forums and in event organisation. Ultimately it will reflect the ability of Aboriginal people to engage in advocacy from a position of being informed and knowledgeable, and to demonstrate leadership. It is essential to recognise that a single issue such as sexually transmitted infections is just one of a multitude of challenges faced by Aboriginal people. Sustained change requires empowerment and social justice, not just program activity within health. Unemployment, homelessness, transport difficulties drug and alcohol misuse and failures in delivery of culturally appropriate services are examples where the need for intersectoral action requires higher level policy to facilitate efforts to control sexually transmitted infections occurring in communities. Our experience is that Aboriginal people are often (appropriately) more focussed on addressing the underlying causes of health disadvantage than any individual health issue, and are most ready to develop partnerships with non-Indigenous people when there is genuine commitment to achieving social justice and health in a much more holistic sense.

## Key lessons learned

• Build effective networks across Aboriginal and non-Aboriginal stakeholders and value all members of the multidisciplinary team. Listen and be responsive to what people are saying. Recognise the importance of building relationships. Ensure there are opportunities to communicate.

• Base arguments and advocacy on data. Ensure that Aboriginal people have access to that information, appreciate its significance, and are given the authority and skills to teach others in their community and to gather new data.

• The power of the story is in the telling and who tells it also matters. Messages from knowledgeable, concerned and passionate Aboriginal experts will have far greater resonance within Aboriginal communities than messages told by well-intentioned whitefellas.

• Properly resource Aboriginal people and organisations to enable meaningful input. Individuals rarely have the luxury of taking hours out of their job to have input into your pet concern.

• Invest in quality training and recognise the value of partnerships. Apprenticeship-style training for Aboriginal people within high functioning organisations committed to Aboriginal health provides two-way learning, and can help those organisations channel their resources to support improvements in Aboriginal health. Build expertise and understanding; work together.

• Recognise the difficulties faced by Aboriginal people who move to the city to start a job, and the isolation that Aboriginal people working in mainstream middle-class organisations often feel. Consider engaging an Aboriginal person who is respected by the relevant parties as a mentor. Encourage Aboriginal employees to take opportunities to network with their colleagues across organisations.

• Don't restrict advocacy efforts to the health sector. Use community networks and take every opportunity to raise the issue and keep the momentum.

• Encourage leadership. Provide information and the space for Aboriginal people with passion to engage with the issue. Step back, but continue to offer encouragement and practical assistance.

• Keep focused and positive. Aboriginal people may feel overwhelmed or torn by the number of health and other issues experienced by their communities, but when supported and well informed, can contribute to a groundswell for change.

## Abbreviations

AIDS: Acquired Immunodeficiency Syndrome; EPS: Explicit Performance Standards; HIV: Human Immunodeficiency Virus; SHBBVP: Sexual Health and Blood-Borne Virus Program; STI: Sexually transmitted infection; WA: Western Australia.

## Competing interests

SCT worked from 1999–2003 in the SHBBVP. HSG is currently working in the SHBBVP and has worked there since 1999. RP worked in the WA state office of the Office of Aboriginal and Torres Strait Islander Health, Department of Health and Ageing from 2002 to 2005. There are no financial or other conflicts of interest.

## Authors' contributions

SCT, HSG and RP were all involved in program planning and funding of health services and in development of strategies to increase discussion of sexual health issues among Aboriginal people. All authors contributed to the drafting of this article and have approved the final version for submission.
